# Population-based histologic analysis of craniopharyngioma demographics and treatment in the US from 2000 to 2020

**DOI:** 10.1007/s11060-025-04988-0

**Published:** 2025-03-05

**Authors:** Kevin E. Agner, Michael C. Larkins

**Affiliations:** 1 The Ohio State University College of Medicine, 370 W. 9th Avenue, Columbus, OH 43210 USA; 2 Department of Emergency Medicine, Boonshoft School of Medicine at Wright State University, 2555 University Blvd, Fairborn, OH USA

**Keywords:** Craniopharyngioma, Cancer histology, Cancer epidemiology, Adamantinomatous craniopharyngioma, Papillary craniopharyngioma, Surgical oncology

## Abstract

**Purpose:**

Craniopharyngiomas (CP) are rare tumors that serve as a source of significant morbidity despite relatively high survival rates. No analysis based on disease histology has been fully explored.

**Methods:**

Patients with CP were identified via the Surveillance, Epidemiology, and End Results (SEER) Program regarding the following ICD-O-3 codes: 9350 (CP, not otherwise specified; NOS), 9351 (adamantinomatous CP; ACP), and 9352 (papillary CP; PCP). Demographic and treatment variables were analyzed via Chi-squared tests and ten-year overall survival (10y OS) was assessed via Cox regression and log-rank analysis.

**Results:**

Variation in 10y OS was seen regarding patient age (*p* < 0.001), race (*p* < 0.001), Grade (*p* = 0.010), stage (*p* < 0.001), and treatment with radiotherapy (*p* < 0.001) with Cox regression analysis among the 2,359 patients identified. ACP or CP, NOS histologies were more frequent among patients < 25 years old (*p* < 0.001), while ACP was more common among male patients (*p* = 0.002) and PCP was less common among Black patients (*p* = 0.002). Univariate survival analysis revealed the ACP and CP, NOS histologies had improved 10y OS with treatment with radiotherapy (*p* < 0.001 and = 0.007, respectively). Finally, surgery was associated with improved 10y OS only among patients with the CP, NOS histology (*p* = 0.007).

**Conclusion:**

No difference in 10-year overall survival was seen regarding histology among diagnosed with craniopharyngioma on multivariate or univariate analysis. Differences in the frequency and survival were found among all three histologies stratified by demographics and treatment. Further investigation into these variables, and among different survival timeframes, is warranted.

**Supplementary Information:**

The online version contains supplementary material available at 10.1007/s11060-025-04988-0.

## Introduction

Although rare and typically benign tumors, craniopharyngiomas (CP) pose significant challenges due to disease site, insidious growth, and potential for recurrence [1]. Arising from embryonic remnants along the craniopharyngeal duct and accounting for 1.2-4% of all intracranial tumors [2], these tumors can cause significant symptoms and morbidity. They primarily affect the central nervous system due to their substantial mass and resulting compression of vital neural structures, particularly the pituitary gland, hypothalamus, and optic chiasm [3, 4]. CPs come in two subtypes: adamantinomatous (ACP) and papillary (PCP) [5]. ACPs, more prevalent in children, are typically cystic with calcifications and contain dark-colored, heterogeneous fluid. PCPs, on the other hand, are found mostly in adults and are mostly solid, contain clear fluid, and lack calcifications. Histologically, ACPs contain wet keratin and stellate reticulum, while PCPs exhibit mature squamous epithelium [5]. Survival rates are relatively high, with five-year survival reported between 83 and 96% and 10-year survival between 65 and 100% [2]. The main management options for CP are gross total resection, or subtotal resection followed by radiation therapy, with both treatment plans resulting in similar OS and progression-free survival according to a recent meta-analysis [6].

Several demographic factors are significantly associated with CP survival in retrospective Surveillance, Epidemiology, and End Results (SEER)-based studies. Race consistently emerged as a risk factor for decreased overall survival (OS), particularly between Black and White populations, suggesting potential disparities in access to care or underlying biological differences [7–9]. Age at diagnosis also has been shown to impact survival, with older patients experiencing decreased OS due to increased comorbidities and compromised physiological conditions [7–9]. Marital status was identified as an independent prognostic factor in middle-aged patients, with married individuals exhibiting better survival compared to single or divorced/separated patients, possibly due to the social and psychological benefits of partnership [7, 9]. While existing research explored prognostic factors influencing survival, including age, race, tumor size, and extent of resection [8, 10, 11], there exists a gap in understanding the histological subtypes and their correlation with epidemiological factors and clinical outcomes.

Teng et al. found no statistically significant difference in OS between ACP and PCP subtypes [8], while Wu et al. did not find any general association between CP histology and OS [9]. Pham et al. reported a marginally significant association between histological subtype and marital status in their univariate analysis (*p* = 0.021), but this link was subsequently contradicted by their multivariate analysis revealing marital status as an independent predictor of mortality [7].

This study aims to explore this gap by conducting a retrospective histological epidemiologic study of CP. Building upon previous research using the SEER database, this study will analyze the specific characteristics of histological subtype and their associations with patient demographics, treatment modalities, and survival outcomes.

## Materials and methods

### Patient identification

Patients with CP were identified via the 17-registry Incidence data set from the Surveillance, Epidemiology, and End Results (SEER) Program [12]. This dataset contains cases diagnosed between 2000 and 2020. Inclusion criteria were the following International Classification of Diseases (ICD)-O-3 codes: 9350/3 (“Craniopharyngioma, malignant”), 9351/0 (“Craniopharyngioma, adamantinomatous, benign”), 9351/1 (“Adamantinomatous craniopharyngioma”), 9352/0 (“Papillary craniopharyngioma, benign”), 9352/1 (“Papillary craniopharyngioma”), and 9352/3 (“Craniopharyngioma, papillary, malignant”). Additionally, only cases with known patient age and positive histology were included.

### Data analysis

Data was analyzed in SPSS (Version 29.0; Armonk, NY: IBM Corp.). A p-value of < 0.05 was used as the cutoff for statistical significance; 95% confidence intervals reported in brackets [95% CI]. Multivariate analysis was conducted via Cox regression based on ten-year overall survival (10y OS). Categorical variables were compared via two-sided Chi-squared tests. Two-sided likelihood ratios were reported if the percentage of cells with an expected count of less than five was greater than 20.0%. Kaplan-Meier (KM) survival curves were generated and compared using log-rank analysis for univariate and subanalysis. Surgical codes were determined based on the 2022 SEER Staging Manual [13].

## Results

### Cohort overview

**Fig. 1:**
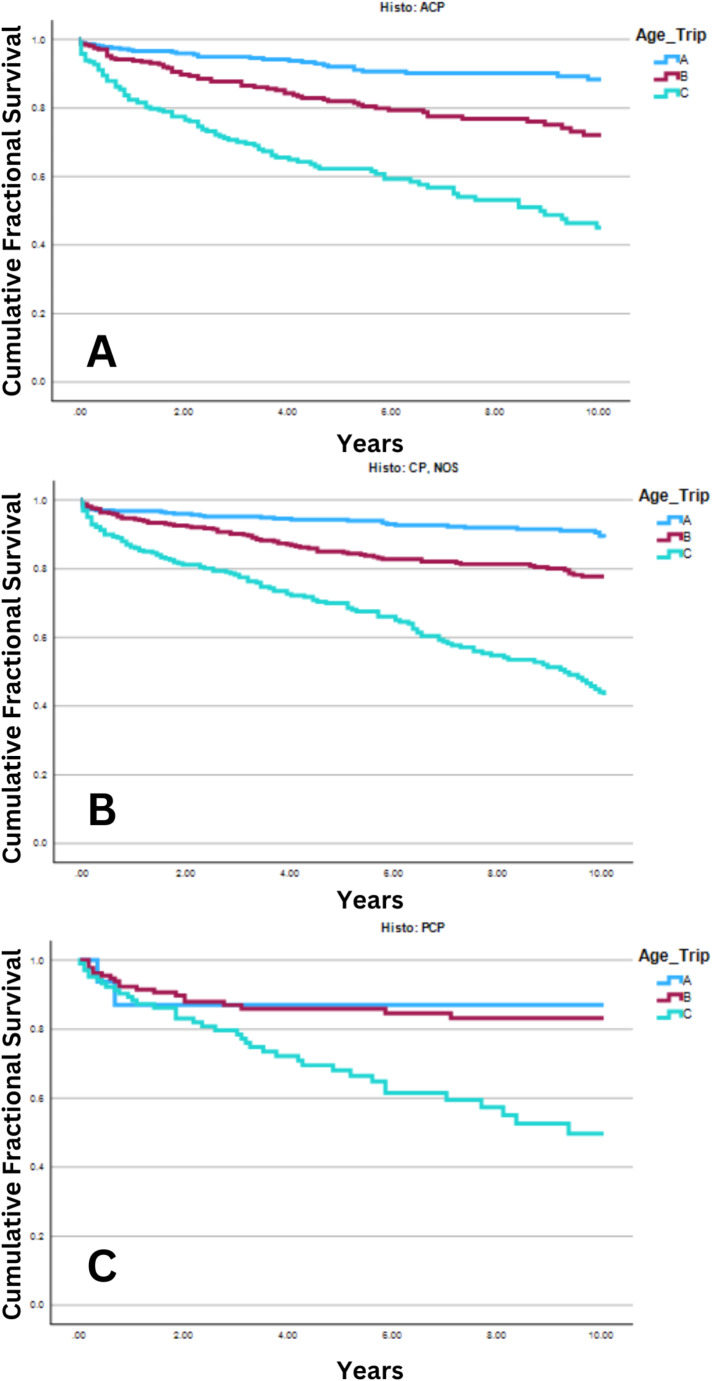
Ten-year Overall Survival (10y OS) of Patients with Craniopharyngioma, Stratified by Histology and Age (*n* = 2,359). 10y OS among patients with craniopharyngioma, as identified from the Surveillance, Epidemiology, and End Results (SEER) Program among patients in the US, diagnosed between 2000 and 2020. Patients were stratified by both age and disease histology; age was divided into three approximately equal groups from the entire cohort. The figure legend in the top right of each panel shows a color-coded legend depicting the age group that corresponds with each survival curve: **A**: 0–24 years of age at diagnosis, **B**: 25–54 years, and **C**: 55–85 + years. Panel A: Patients with the adamantinomatous craniopharyngioma histology (ACP). Patients 0–24 years had increased 10y OS (88.3% [83.9%,92.7%]) compared to both the 25–54 year (72.1% [65.5%,78.7%]) and 55–85 + year groups (45.0% [36.4%,53.6%]; *p* < 0.001). Panel B: Patients with the “Not Otherwise Specified (NOS)” craniopharyngioma histology (CP, NOS). Patients 0–24 years had increased 10y OS (89.0% [85.4%,92.6%]) compared to both the 25–54 year (77.7% [73.1%,82.3%]) and 55–85 + year groups (43.2% [35.8%,50.6%]; *p* < 0.001). Panel C: Patients with papillary craniopharyngioma histology (PCP). Patients 0–24 years had increased 10y OS (87.1% [69.9%,100%]) compared to both the 25–54 year (83.2% [76.0%,90.4%]) and 55–85 + year groups (49.7% [36.5%,62.9%]; *p* < 0.001)

Two-thousand three hundred and fifty-nine patients were identified with CP. Median five-year age bracket was 40–44 years; case incidence by age can be seen in Fig. 1SM of the Supplementary Materials. Case distribution by age can be seen to follow a roughly bimodal incidence, with peaks in the five- to nine-year and 50-to 54-year age brackets.

A summary of patient demographic, disease characteristic, and treatment information can be found in Table 1. Disease Grade was consolidated across multiple SEER coding variables; the schema for this was derived from the Grade Coding Instructions and Table [14]. A table showing the converted values can be found in Table 1 of Appendix A. Determination of the surgical procedure type was based on the breakdown presented in Teng et al. [8], with the following modifications: for all sites, code 90 (corresponding to “Surgery Not Otherwise Specified”) was grouped with codes 00 and 20. Additionally, for sites C70.0, C71.0-71.9, and C72.0-72.9, only code 21 (corresponding to “Subtotal resection of tumor, lesion or mass in brain “) was included among subtotal resection (STR) cases. Similarly, codes 30, 40, and 60 were considered examples of gross total resection (GTR) among cases with primary sites 75.1–75.3. These changes were made to better approximate the nature of the surgery being performed (e.g. partial resection of a brain lobe was considered to have been conducted with the goal of resecting the entirety of a tumor and therefore would be classified as GTR, etc.). A table showing the breakdown for consolidation of surgical codes into STR, GTR, etc. can be found in Table 2 of Appendix A.

**Table 1:** Demographic, disease characteristic, and treatment information for patients diagnosed with craniopharyngioma

Variable	Number (% of Cohort; *n* = 2,359)
*Age at Diagnosis*	
0–24 Years	792 (33.6%)
25–54 Years	874 (37.0%)
55–85+	693 (29.4%)
*Sex*	
Male	1233 (52.3%)
Female	1126 (47.7%)
*Race*	
Black	376 (15.9%)
White	1698 (72.0%)
Other*	255 (10.8%)
Unknown	30 (1.3%)
*Histology*	
CP, NOS (ICD-O-3: 9350)	1153 (48.9%)
ACP (ICD-O-3: 9351)	951 (40.3%)
PCP (ICD-O-3: 9352)	255 (10.8%)
*Grade*	
1	357 (15.1%)
2	1 (0.0%)
3	1 (0.0%)
4	1 (0.0%)
Unknown	1999 (84.7%)
*Stage*	
In Situ	1 (0.0%)
Localized	5 (0.2%)
Regional	3 (0.1%)
Distant	0 (0.0%)
Unknown	2350 (99.6%)
*Radiotherapy*	
No Radiation/Unknown	2182 (75.8%)
Radiation	572 (24.2%)
*Chemotherapy*	
No Chemotherapy/Unknown	2343 (99.3%)
Chemotherapy	16 (0.7%)
*Surgical Procedure*	
None/Unknown/Biopsy/Not Otherwise Specified	603 (25.6%)
STR	661 (28.0%)
GTR	1095 (46.4%)

Patient demographic, disease characteristic, and treatment information for patients diagnosed with craniopharyngioma in the US between 2000 and 2020, as identified via the surveillance, epidemiology, and end results (SEER) program

*Includes patients coded as American Indian/Alaskan Native and Asian/Pacific Islander

CP: Craniopharyngioma; NOS: Not Otherwise Specified; ACP: Adenomatous Craniopharyngioma; PCP: Papillary Craniopharyngioma; STR: Subtotal Resection; GRT: Gross Total Resection

**Table 2:** Cox regression analysis of Ten-year overall survival among patients with craniopharyngioma (*n* = 2,359)

Variable	Hazard Ratio	*P*-value
*Age at Diagnosis*	N/A	< 0.001
25–54 Years vs. 55–85 + and 0–24 Years	2.23	< 0.001
55–85 + Years vs. 25–54 and 0–24 Years	5.99	< 0.001
*Sex (Male vs. Female)*	1.17	0.08
*Race*	N/A	< 0.001
White vs. Black and Other*	10.01	0.90
Black vs. White and Other*	21.68	0.86
*Histology*	N/A	0.25
CP, NOS vs. ACP and PCP	0.85	0.10
PCP vs. ACP and CP, NOS	0.88	0.40
*Grade*	N/A	0.010
*Stage*	N/A	< 0.001
Regional vs. All Other Stages	14.54	0.033
*Radiotherapy (Yes vs. No)*	0.64	< 0.001
*Chemotherapy (Yes vs. No)*	1.09	0.90
*Surgery*	N/A	0.50
None/Unknown/Biopsy/Not Otherwise Specified vs. STR and GTR	1.13	0.27

Results from multivariate Cox regression analysis. overall p-value is listed for categorical variables with more than two categories, while hazard ratios and the associated p-values are provided for comparisons with two categories. Significance was seen with patient age (*p* < 0.001), race (*p* < 0.001), disease grade (*p* = 0.010), stage (*p* < 0.001), and treatment with radiotherapy (*p* < 0.001)

*Includes patients coded as American Indian/Alaskan Native and Asian/Pacific Islander

CP: Craniopharyngioma; NOS: Not Otherwise Specified; ACP: Adenomatous Craniopharyngioma; PCP: Papillary Craniopharyngioma; STR: Subtotal Resection; GTR: Gross Total Resection

### Multivariate analysis

Cox regression analysis with respect to 10-year overall survival (10y OS) was performed for the aforementioned demographic, disease characteristic, and treatment variables. Results can be found in Table 2. Patient age at diagnosis with CP was divided into three approximately equal groups to allow for strong statistical comparison. In general, younger patients (aged 0–24 years) were found to have increased survival compared to all other groups (*p* < 0.001). Furthermore, variations in 10y OS with respect to race, disease Grade, stage, and treatment with radiotherapy (hazard ratio (HR) = 0.64; *p* < 0.001) were found. No variation was seen regarding CP histology among the three histologies assessed (*p* = 0.21).

### Chi-squared analysis with histology

Association between CP histology and the other collected SEER variables was conducted using Chi-squared testing. Results can be found in Table 3. It can be seen that in general patients < 25 years at diagnosis were more likely to be diagnosed with either ACP or CP, NOS, as opposed to PCP (*p* < 0.001). Male patients (41.4%) were more likely to be diagnosed with ACP than female patients (39.2%), while the opposite was true of the PCP histology (12.6% vs. 8.9%, respectively; *p* = 0.002). White patients (12.0%) and those coded as Other (12.9%) were more likely to be diagnosed with PCP as compared to black patients (4.3%; *p* = 0.002).

**Table 3:** Chi squared univariate analysis among patients with craniopharyngioma, stratified by disease histology (*n* = 2,359)

Variable	Histology (% of cohort)			*P*-value
	ACP	CP, NOS	PCP	
*Age*				< 0.001
0–24 Years	369 (15.6%)	406 (17.2%)	17 (0.7%)	
25–54 Years	318 (13.5%)	422 (17.9%)	134 (5.7%)	
55–85+	264 (11.2%)	325 (13.8%)	104 (4.4%)	
*Sex*				0.002
Female	441 (18.7%)	585 (24.8%)	100 (4.2%)	
Male	510 (21.6%)	568 (24.1%)	155 (6.6%)	
*Race*				0.002
White	674 (28.6%)	821 (34.8%)	203 (8.6%)	
Black	159 (6.7%)	201 (8.5%)	16 (0.7%)	
Other*	106 (4.5%)	116 (4.9%)	33 (1.4%)	
Unknown	12 (0.5%)	15 (0.6%)	3 (0.1%)	
*Grade*				< 0.001
Unknown (0)	738 (31.3%)	1061 (45.0%)	200 (8.5%)	
1	213 (9.0%)	89 (3.8%)	55 (2.3%)	
2	0 (0.0%)	1 (0.0%)	0 (0.0%)	
3	0 (0.0%)	1 (0.0%)	0 (0.0%)	
4	0 (0.0%)	1 (0.0%)	0 (0.0%)	
*Stage*				0.23
In Situ	0 (0.0%)	0 (0.0%)	1 (0.0%)	
Local	1 (0.0%)	4 (0.2%)	0 (0.0%)	
Regional	1 (0.0%)	1 (0.0%)	1 (0.0%)	
Unknown	949 (40.2%)	1148 (48.7%)	253 (10.7%)	
*Radiation*				0.82
No Radiation	738 (31.3%)	884 (37.5%)	194 (8.2%)	
Radiation	213 (9.0%)	269 (11.4%)	61 (2.6%)	
*Chemotherapy*				0.11
No Chemotherapy	948 (40.2%)	1141 (48.4%)	254 (10.8%)	
Chemotherapy	3 (0.1%)	12 (0.5%)	1 (0.0%)	
*Surgery*				0.40
None	224 (9.5%)	307 (13.0%)	72 (3.1%)	
STR	269 (11.4%)	325 (13.8%)	67 (2.8%)	
GTR	458 (19.4%)	521 (22.1%)	116 (4.9%)	

Results from two-sided Chi-squared analysis of patients diagnosed with CP stratified by disease histology (ACP, CP, NOS, and PCP). Significance was seen with patient age (*p* < 0.001), sex (*p* = 0.002), race (*p* = 0.002), and grade (*p* < 0.001)

*Includes patients coded as American Indian/Alaskan Native and Asian/Pacific Islander

CP: Craniopharyngioma; NOS: Not Otherwise Specified; ACP: Adenomatous Craniopharyngioma; PCP: Papillary Craniopharyngioma; STR: Subtotal Resection; GTR: Gross Total Resection

### Univariate histologic survival analysis

The results of 10y OS KM curve analysis can be found in Table 4. No difference in 10y OS was seen between histologic subtypes (*p* = 0.17). Stratifying by both histology and age (divided into three approximately equal groups: 0–24 Years, 25–54 Years, and 55–85 + Years) revealed patients 0–24 years of age at diagnosis had increased 10y OS compared to both patients aged 25–54 years and 55–85 + years (*p* < 0.001; see Fig. 1). Similarly, for both the ACP and CP, NOS histologies, patients 25–54 years at diagnosis had increased 10y OS compared to those 55–85 + years (*p* < 0.001). However, with the PCP histology no difference was seen between these two age groups (*p* = 0.84).

**Table 4:** Kaplan-Meier 10-year overall survival (10y OS) univariate analysis among patients with craniopharyngioma, stratified by disease histology (*n* = 2,359)

Variable	10y OS by Histology, % [95% CI]		
	ACP	CP, NOS	PCP
*Overall*	71.0% [67.0%,75.0%]	73.1% [69.9%,76.3%]	69.7% [62.5%,76.9%]
	*p* = 0.17		
*Age*			
0–24 Years	88.3% [83.9%,92.7%]	89.0% [85.4%,92.6%]	87.1% [69.9%,100%]
25–54 Years	72.1% [65.5%,78.7%]	77.7% [73.1%,82.3%]	83.2% [76.0%,90.4%]
55–85+	45.0% [36.4%,53.6%]	43.2% [35.8%,50.6%]	49.7% [36.5%,62.9%]
	*p* < 0.001	*p* < 0.001	*p* < 0.001
*Sex*			
Female	71.6% [66.0%,77.2%]	74.5% [70.3%,78.7%]	69.8% [58.4%,81.2%]
Male	70.5% [65.1%,75.9%]	71.6% [67.0%,76.2%]	69.9% [60.9%,78.9%]
	*p* = 0.70	*p* = 0.40	*p* = 0.64
*Race*			
White	75.4% [71.0%,79.8%]	75.4% [71.8%,79.0%]	71.3% [63.5%,79.1%]
Black	49.3% [39.1%,59.5%]	56.5% [47.9%,65.1%]	52.9% [22.9%,82.9%]
Other*	75.1% [63.7%,86.5%]	82.5% [73.3%,91.7%]	74.9% [57.9%,91.9%]
Unknown	0%	0%	0%
	*p* < 0.001	*p* < 0.001	*p* = 0.16
*Grade*			
Unknown (0)	72.4% [68.4%,76.4%]	73.6% [70.4%,76.8%]	69.0% [61.4%,76.6%]
1	51.5% [33.3%,69.7]	56.8% [32.6%,81.0%]	73.7% [52.9%,94.5%]
2	N/A**	N/A**	N/A**
3	N/A**	0%	N/A**
4	N/A**	N/A**	N/A**
	*p* = 0.20	*p* > 0.001	*p* = 0.53
*Stage*			
In Situ	N/A**	N/A**	N/A**
Local	N/A**	0%	N/A**
Regional	N/A**	0%	0%
Unknown	71.0% [67.0%,75.0%]	73.1% [69.9%,76.3%]	69.9% [62.7%,77.1%]
	*p* = 0.86	*p* = 0.026	*p* < 0.001
*Radiation*			
No Radiation	67.7% [63.1%,72.3%]	71.5% [67.9%,81.3%]	69.7% [61.5%,77.9%]
Radiation	82.4% [75.4%,89.4%]	78.4% [72.0%,84.8%]	70.3% [56.1%,84.5%]
	*p* < 0.001	*p* = 0.007	*p* = 0.64
*Chemotherapy*			
No Chemotherapy	71.1% [67.1%,75.1%]	72.9% [69.7%,76.1%]	70.1% [62.9%,77.3%]
Chemotherapy	66.7% [12.3%,100%]	100%	0%
	*p* = 0.61	*p* = 0.12	*p* = 0.052
*Surgery*			
None	66.2% [57.8%,74.6%]	65.5% [58.7%,72.3%]	75.9% [63.5%,88.3%]
STR	69.9% [61.9%,77.9%]	79.4% [74.0%,84.8%]	58.8% [42.5%,74.5%]
GTR	74.0% [68.8%,79.2%]	73.5% [68.9%,78.1%]	72.2% [62.4%,82.0%]
	*p* = 0.28	*p* = 0.007	*p* = 0.10

Results from univariate 10y OS analysis of patients diagnosed with CP stratified by disease histology (ACP, CP, NOS, and PCP). For comparisons within a histologic group, the p-value representing the log-rank comparison of all categories in a variable is listed underneath all categories within the respective column

*Includes patients coded as American Indian/Alaskan Native and Asian/Pacific Islander

**No cases listed under this category

CP: Craniopharyngioma; NOS: Not Otherwise Specified; ACP: Adenomatous Craniopharyngioma; PCP: Papillary Craniopharyngioma; STR: Subtotal Resection; GTR: Gross Total Resection

Regarding sex, no difference was seen between male and female patient 10y OS for any CP histology (*p* = 0.70 for ACP, 0.40 for CP, NOS, and *p* = 0.64 for PCP). Concerning race, no difference was seen among patients diagnosed with the PCP variant of CP, however Black patients with the ACP and CP, NOS histologies had decreased 10y OS compared to both White and Other (including Asian and Pacific Islander) patients (*p* < 0.001 for both comparisons). Comparison concerning Grade and stage were limited due to the majority of patients (84.7% and 99.6%, respectively; see Table 1) having unknown information for these variables.

Regarding treatment, patients with both the ACP and CP, NOS histologies of CP had improved 10y OS with treatment with RT (*p* < 0.001 and = 0.007, respectively), while those with PCP did not have survival benefit from RT (*p* = 0.64). No survival benefit was seen with chemotherapy among any of the CP histologies. Finally, surgery was associated with improved 10y OS only among patients with the CP, NOS histology (*p* = 0.007). This was mainly driven by the improved survival seen between patients that did not undergo surgery (10y OS = 65.5%) compared to those that underwent subtotal resection (10y OS = 79.4%). Direct comparison between these two groups was significant (*p* = 0.001), as was comparison among patients with PCP (*p* = 0.046), while analysis among patients with ACP had no difference in 10y OS (*p* = 0.49).

## Discussion

This study investigated the association between histological subtypes of CP and patient demographics, treatment modalities, and 10y OS using a retrospective cohort of CP cases diagnosed between 2000 and 2020 in the US. Our analysis revealed a clear age-related difference in survival, with younger patients experiencing better survival outcomes. For instance, the youngest age group (0–24 years) exhibited 10y OS of 88.3% for adamantinomatous craniopharyngioma (ACP), 89.0% for craniopharyngioma, not otherwise specified (CP, NOS), and 87.1% for papillary craniopharyngioma (PCP); in contrast, the oldest age group (55–85+) had much lower survival rates of 45.0%, 43.2%, and 49.7% respectively (*p* < 0.001, see Table 4; Fig. 1). This finding is consistent with previous studies, suggesting that increased comorbidities and compromised physiological conditions in older patients contribute to their decreased OS [7–9]. Furthermore, patients aged 25–54 years at diagnosis had a significantly higher hazard ratio for 10y OS compared to 0-24-year-olds, and 55–85 + year-olds exhibited the highest risk of all age groups (HR = 2.23, *p* < 0.001, and HR = 5.99, *p* < 0.001, respectively; see Table 2).

No significant difference in 10y OS was observed between the three CP histological subtypes analyzed in this study (ACP, PCP, and CP, NOS) on either multivariate (*p* = 0.25; see Table 2) or univariate (*p* = 0.17; see Table 4). This may be secondary to the age distribution of these subtypes (see Fig. 1). PCP, being more prevalent in older patients (4.4% of the cohort, *p* < 0.001; see Table 3), may reflect the overall decreased survival seen in this age group rather than being inherently more aggressive. This finding is also in line with previous reports by Teng et al. and Wu et al., both of which found no significant difference in OS between ACP and PCP subtypes [8, 9]. Furthermore, in the univariate analysis of PCP, there was uniquely no observable difference in 10y OS among patients aged 25–54 and 0–24, although there was a marked difference between the 55–85 + year old group, as is seen with the other histologic subtypes, as well (*p* < 0.001; Fig. 1). This may indicate that the PCP 25–54-year-old group has increased overall survival compared to patients over 55, but the exact mechanism underlying this is unknown. The reason for this disparity in the effect of age on OS is unclear, but may be related to a smaller sample size for the PCP group or different disease biology, and further studies are needed to investigate the effects of age on PCP outcomes. The lack of an effect of age on the multivariate analysis is likely due to the overall power of the entire cohort overriding the statistical differences seen when stratifying by histology, a situation which is particularly relevant in the PCP subtype as it only accounts for roughly 10% of the total study group. On the other hand, this may be reflective of the increased mortality associated with surgery and oncologic treatment among those with increased age in general.

Interestingly, Black patients with ACP and CP, NOS were found to have significantly decreased 10y OS of 49.3% and 56.5% respectively, compared to the White (75.4% and 75.4%) and Other patients, which include those listed as Asian/Pacific Islander and American Indian/Alaska Native (75.1% and 82.5%, *p* < 0.001; see Table 4). This finding is consistent with previous research demonstrating racial disparities in CP survival [7–9], highlighting the need for further investigation to understand the underlying causes, be it access to care, social determinants of health, or underlying biological differences. Additionally, Black patients were less likely to be diagnosed with PCP, with only 0.7% diagnosed in this cohort compared to other races (*p* = 0.002; see Table 3).

Furthermore, our study revealed that both ACP and CP, NOS patients treated with radiotherapy (RT) had an associated increased 10y OS, while PCP patients showed no such benefit (see Table 4). The lack of benefit from RT in the PCP group may be due to the more aggressive nature of this subtype, a finding not previously reported in the literature. However, it’s important to note that the smaller number of PCP cases in our cohort (10.8% of the cohort; see Table 1) might limit the generalizability of this finding and may have decreased the statistical power to observe the true effect of radiation therapy on this subgroup. For instance, patients with ACP and CP, NOS who received radiation therapy had a 10y OS of 82.4% [75.4%, 89.4%] and 78.4% [72.0%, 84.8%], respectively, compared to those who did not receive RT, which was 67.7% [63.1%, 72.3%] and 71.5% [67.9%, 81.3%], respectively (see Table 4). These differences in 10y OS between the radiation treatment groups were found to be significant for both ACP (*p* < 0.001) and CP, NOS (*p* = 0.007). It is important to note that one specific area of advancement with treatment of PCP in particular is the use of targeted therapy with DRAF/MEK inhibitors to target key mutations specific to PCP [15]. This was outside of the scope of this project but remains an area of future study.

Regarding surgical procedures, our findings suggest that STR appears to be non-inferior to GTR, particularly for CP, NOS patients, with STR even demonstrating improved 10y OS comparatively (see Table 4). This finding is consistent with recent meta-analyses reporting similar OS and progression-free survival rates between STR and GTR, suggesting that the extent of surgical resection alone may not be the sole determinant of survival [6]. Our analysis corroborates the idea that GTR may not necessarily be associated with improved survival compared to no surgery. However, it is important to note that our analysis does not account for the potential increased morbidity and mortality associated with GTR, which could contribute to the observed lack of survival advantage [16].

The limited information on stage and grade for the majority of our cohort might be due to the curative nature of surgical resection, suggesting that staging and grading information may be less relevant for patients who undergo successful tumor removal. The SEER dataset has substantial missing data for disease grade and stage variables; 84.7% and 99.6% respectively, of patients were missing these pieces of information. This finding aligns with previous SEER studies, in which limited information on disease Grade and stage were found [17, 18]. With similarly low frequency, chemotherapy was administered in merely 0.7% of cases (see Table 1), aligning with the standard practice of surgical resection with radiation in the majority of patients [16]. However, it is our understanding that this is the first SEER analysis of CP to investigate the effect of chemotherapy on survival.

While this study offers valuable insights, it is essential to acknowledge its limitations. The reliance on a multi-center database may constrain the depth of information on individual patient-level factors. Moreover, the absence of data on treatment-associated morbidity and patient frailty, both of which significantly influence treatment decisions, is a notable limitation. Additionally, the SEER Program does not provide specific details on the doses or agents used in treatments, nor does it include information regarding the type of chemotherapy and related specifics. Further investigation into these areas, as well as the differences in over survival timeframes and with cause-specific survival, is warranted.

## Conclusion

No difference in 10-year overall survival was seen among adamantinomatous, not otherwise specified (NOS), and papillary histologies in patients diagnosed with craniopharyngioma on multivariate or univariate analysis. Papillary craniopharyngioma was found to occur more frequently among older, male, and White or Asian/Pacific Islander/American Indian/Alaska Native patients, and contrary to the other two histologies, treatment of this histology with radiotherapy was not associated with increased survival. Subtotal resection was noninferior to gross total resection and demonstrated survival benefit among patients with either NOS or papillary histological variants. To date this is the largest analysis of craniopharyngiomas in the United States and also the first to present a mainly histologic analysis of this tumor.

## Electronic supplementary material

Below is the link to the electronic supplementary material.

Supplementary Material 1

## Data Availability

No datasets were generated or analysed during the current study.
