# Supplementary material for: Population-based histologic analysis of craniopharyngioma demographics and treatment in the US from 2000 to 2020
Source: J Neurooncol. 2025 Mar 5;173(2):343–51. doi: 10.1007/s11060-025-04988-0 (PMC12106490; doi:10.1007/s11060-025-04988-0)
Supplement: Supplementary file 1 — Supplementary Material 1 [file 11060_2025_4988_MOESM1_ESM.docx]

**Supplementary Materials:**

**Figure 1SM:** Distribution of Craniopharyngioma Diagnoses by Age (n = 2,359)


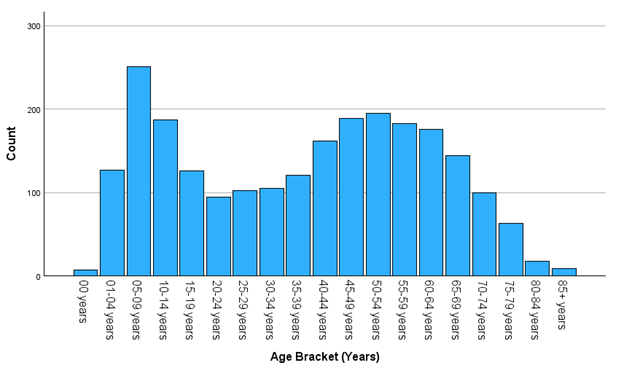


**Figure 1 Caption:** Incidence of craniopharyngioma by five-year age bracket, as reported in data from the Surveillance, Epidemiology, and End Results (SEER) Program among patients in the US, diagnosed between 2000 and 2020.

**Table 1:** Consolidation of Craniopharyngioma Cases based on Grade

| Consolidated Grade | SEER Grading Variable | SEER Grading Variable Value |
| --- | --- | --- |
| 0 | Grade Recode Through 2017 | Unknown |
| 0 | Pathologic Grade 2018 | 9 |
| 1 | Grade Recode Through 2017 | Well-differentiated (Grade I) |
| 1 | Pathologic Grade 2018 | 1 |
| 1 | Pathologic Grade 2018 | L |
| 2 | Grade Recode Through 2017 | Moderately Differentiated (Grade II) |
| 3 | Grade Recode Through 2017 | Poorly Differentiated (Grade III) |
| 4 | Grade Recode Through 2017 | Undifferentiated (Grade IV) |

**Table 1 Caption:** Table depicting how cases were recoded from SEER Grade variables into a consolidated Grade variable.

**Table 2:** Consolidation of Craniopharyngioma Cases based on Surgical Procedure

| *C70.0, C71.0-71.9, C72.0-72.9* | |
| --- | --- |
| **Consolidated Surgery Variable Category** | **SEER Surgical Codes Included** |
| None/Unknown/Biopsy/Not Otherwise Specified | 0, 20, 90 |
| Subtotal Resection | 21 |
| Gross Total Resection | 22, 30, 40, 55 |
| *C75.1-75.3* | |
| **Consolidated Surgery Variable Category** | **SEER Surgical Codes Included** |
| None/Unknown/Biopsy/Not Otherwise Specified | 0, 27, 90, 99 |
| Subtotal Resection | 10, 20-26, 50 |
| Gross Total Resection | 30, 40, 60 |

**Table 2 Caption:** Table depicting how cases were recoded from SEER surgery codes into a consolidated surgery variable.
